# Identifying the Science and Technology Dimensions of Emerging Public Policy Issues through Horizon Scanning

**DOI:** 10.1371/journal.pone.0096480

**Published:** 2014-05-30

**Authors:** Miles Parker, Andrew Acland, Harry J. Armstrong, Jim R. Bellingham, Jessica Bland, Helen C. Bodmer, Simon Burall, Sarah Castell, Jason Chilvers, David D. Cleevely, David Cope, Lucia Costanzo, James A. Dolan, Robert Doubleday, Wai Yi Feng, H. Charles J. Godfray, David A. Good, Jonathan Grant, Nick Green, Arnoud J. Groen, Tim T. Guilliams, Sunjai Gupta, Amanda C. Hall, Adam Heathfield, Ulrike Hotopp, Gary Kass, Tim Leeder, Fiona A. Lickorish, Leila M. Lueshi, Chris Magee, Tiago Mata, Tony McBride, Natasha McCarthy, Alan Mercer, Ross Neilson, Jackie Ouchikh, Edward J. Oughton, David Oxenham, Helen Pallett, James Palmer, Jeff Patmore, Judith Petts, Jan Pinkerton, Richard Ploszek, Alan Pratt, Sophie A. Rocks, Neil Stansfield, Elizabeth Surkovic, Christopher P. Tyler, Andrew R. Watkinson, Jonny Wentworth, Rebecca Willis, Patrick K. A. Wollner, Kim Worts, William J. Sutherland

**Affiliations:** 1 Centre for Science and Policy, University of Cambridge, Cambridge, United Kingdom; 2 Sciencewise, Harwell, Didcot, United Kingdom; 3 The Babraham Institute, Cambridge, United Kingdom; 4 School of the Physical Sciences, University of Cambridge, Cambridge, United Kingdom; 5 Nesta, London, United Kingdom; 6 Department for Business, Innovation and Skills, London, United Kingdom; 7 Involve, London, United Kingdom; 8 Ipsos MORI, London, United Kingdom; 9 Science, Society and Sustainability (3S) Group, School of Environmental Sciences, University of East Anglia, Norwich, United Kingdom; 10 Clare Hall, Cambridge, United Kingdom; 11 NanoDTC, University of Cambridge, Cambridge, United Kingdom; 12 Faculty of Education, University of Cambridge, Cambridge, United Kingdom; 13 Oxford Martin Programme on the Future of Food, University of Oxford, Oxford, United Kingdom; 14 Department of Psychology, University of Cambridge, Cambridge, United Kingdom; 15 RAND Europe, Cambridge, United Kingdom; 16 The Royal Society, London, United Kingdom; 17 Department of Biochemistry, University of Cambridge, Cambridge, United Kingdom; 18 Public Health England, London, United Kingdom; 19 Department of Geographical Sciences, University of Bristol, Bristol, United Kingdom; 20 Pfizer Ltd, Kent, United Kingdom; 21 Department for Environment, Food and Rural Affairs, London, United Kingdom; 22 Natural England, London, United Kingdom; 23 University of Bristol, Bristol, United Kingdom; 24 Cranfield University, Cranfield, United Kingdom; 25 Department of Chemistry, University of Cambridge, Cambridge, United Kingdom; 26 Understanding Animal Research, London, United Kingdom; 27 Department of History and Philosophy of Science, University of Cambridge, Cambridge, United Kingdom; 28 The Royal Academy of Engineering, London, United Kingdom; 29 Cabinet Office, London, United Kingdom; 30 Cambridge Centre for Climate Change Mitigation Research (4CMR), Department of Land Economy, University of Cambridge, Cambridge, United Kingdom; 31 Defence Science and Technology Laboratory, Salisbury, United Kingdom; 32 Keble College, Oxford, United Kingdom; 33 Pembroke College, Cambridge, United Kingdom; 34 University of Southampton, Southampton, United Kingdom; 35 Home Office, London, United Kingdom; 36 Ministry of Defence, London, United Kingdom; 37 Government Office for Science, London, United Kingdom; 38 Parliamentary Office of Science and Technology, London, United Kingdom; 39 School of Environmental Sciences, University of East Anglia, Norwich, United Kingdom; 40 Green Alliance, London, United Kingdom; 41 Engineering Design Centre, Department of Engineering, University of Cambridge, Cambridge, United Kingdom; 42 Department of Zoology, University of Cambridge, Cambridge, United Kingdom; Max Planck Society, Germany

## Abstract

Public policy requires public support, which in turn implies a need to enable the public not just to understand policy but also to be engaged in its development. Where complex science and technology issues are involved in policy making, this takes time, so it is important to identify emerging issues of this type and prepare engagement plans. In our horizon scanning exercise, we used a modified Delphi technique [1]. A wide group of people with interests in the science and policy interface (drawn from policy makers, policy adviser, practitioners, the private sector and academics) elicited a long list of emergent policy issues in which science and technology would feature strongly and which would also necessitate public engagement as policies are developed. This was then refined to a short list of top priorities for policy makers. Thirty issues were identified within broad areas of business and technology; energy and environment; government, politics and education; health, healthcare, population and aging; information, communication, infrastructure and transport; and public safety and national security.

## Introduction

It is now widely accepted that effective public policy development requires not simply that the public understands policy proposals but also that the public be actively engaged, from the outset, in the design and formulation of those proposals [2], [3]. Where policy-making is confronted with complex, challenging or uncertain science and technology, meaningful engagement of any kind will inevitably demand both considerable preparation and an enhanced level of public dialogue. Whilst it is important that such dialogue serves to promote better public understanding of relevant science and technology, this should not be its central purpose. Instead, the fundamental objective of public engagement should be to enhance the sensitivity of all actors – scientists, policy-makers and wider publics alike – to the inherently social, ethical and value-based dimensions of particular problems and policy proposals. Engagement of this kind thus fulfils a ‘normative’ rationale (allowing publics to have a say on issues that affect them), an ‘instrumental’ rationale (facilitating learning on the part of citizens about the world in which they live) *and* a ‘substantive’ rationale (improving the quality of policy decisions by bringing new forms of knowledge to bear on the policy-making process) [4]. Under this model publics are actively engaged, in short, in debates over the choice of *ends* as well as *means* in the sphere of public policy [5].

In the past, procedural shortcomings of public engagement, as well as reluctance amongst some experts to consider fully the value-laden social and ethical dimensions of complex policy problems, have fostered suspicion and distrust of scientists and policy-makers on the part of the public. Such suspicions were clearly evident in the discussions surrounding the UK Government’s efforts to gain public assent for the commercial development of genetically modified crops in the early 2000s for instance [6], just as today they can be seen in debates over the use of hydraulic fracturing (or ‘fracking’) by fossil fuel companies seeking to access shale gas reserves. In both cases, debate might arguably have been less polemical and more constructive had public engagement efforts been staged ‘upstream’ of the policy process [7], been as inclusive as possible, and refused to privilege any one particular type of knowledge or perspective over others (see for instance [8]).

That said, politicians are elected for comparatively short terms of office and required to address disputed, value-laden, complex, interacting and long-term challenges; unless the process and results of activities are relevant and sensitive to institutional decision-making and organisational structures, the benefits that public engagement can deliver will be limited [9]. In the UK context, the ScienceHorizons programme (http://www.sciencewise-erc.org.uk/cms/sciencehorizons/) provides a useful example of early upstream engagement; however, because the issues addressed in this programme were so far upstream, there were significant problems in developing and demonstrating direct links between the discussions in the project and their policy impact [10]. Insights drawn from early engagement might become out-dated or irrelevant if participation is confined to one-off exercises and no capacity for reflexivity is built into science and technology institutions and decision processes. However, where decision-making can incorporate mechanisms to reflect on-going public dialogue, these can be used to help decision makers identify emerging issues and gain insight into how they might be better characterised and tackled. Further, the emergence of novel issues from public dialogue can usefully influence the choice of topics for research and innovation and the trajectory of science and technology development. Where relevant agendas or policies have yet to be developed, more effective responses to the outcomes of engagement could be facilitated by the early involvement of politicians.

The aim of this paper is to identify future issues involving science and technology that potentially require public dialogue to improve policy development. It reports on the outcomes of a workshop at the University of Cambridge on 26/27 March 2013 and represents a first attempt to document the science and technology dimensions of emerging public policy issues.

## Materials and Methods

The Delphi technique (see e.g. [11]) has been used since the 1950s [12] as process of forecasting using interactive expert discussion. Experts are asked to provide a confidential assessment of a problem and are then presented with the summary statistics, on which they then contribute to an anonymous discussion. Those whose scores on an issue are outliers should either change their vote to conform or should justify why their view is correct; there is no need for individuals to conform. The process is repeated a number of times to reach a combined view to which all can sign up. This approach is increasingly being recommended for use on a range of issues [13], [14] and has been subject to considerable discussion [15]. Experiments have shown that the Delphi technique is more effective than simply using individual experts [16].

Sutherland et al [1] have developed what is, in effect, a reduced and rapid form of Delphi exercise, although with some characteristics of focus groups [17], which has previously been successfully applied to a range of issues including those emerging in conservation science [18], [19], [20], agriculture [21], science policy [22], and poverty reduction [23]. The approach facilitates a group of people with broad knowledge across the subject area under study, each with specialist expertise in one or a few subsets of issues within it, to evolve a broad set of emerging issues and then to refine this in debate to those seen as most salient with respect to the purpose of the exercise. We chose this approach as being appropriate to our task and proportionate in its use of specialist resources.

In this case, we used a three-stage approach. Firstly, 930 public policy professionals were each invited to identify 3 emerging policy issues. These professionals were selected from a database of 8000 people with an interest in science and public policy held by the Centre of Science and Policy’s (CSaP). A purposive sample was taken to represent people working at middle to senior management levels on public policy (i.e. with enough experience to have an informed overview of emerging trends). The sample included people working in government, Parliament and the civil service in the UK but also included some other countries and the EU and from the private and higher education sectors. Of these 930, 8% (79 people with 25 separate affiliations) responded and supplied an initial list of 131 policy issues, which was refined to 100 (not an intended target) after identification of overlaps and duplications.

Secondly, we mailed a further 352 people from the science community, also drawn from the CSaP database (i.e. scientists with an interest in and experience of policy); they were mostly at middle to senior management levels, and mostly from the UK. They were invited to suggest emerging S&T challenges that would have an affect on how those policy issues could be addressed. Some 7% (25 people) identified some 648 S&T developments that intersected with these 100 policy issues. These were tabulated and re-sent to all members of this group to prioritise according to which issues were most “likely to be challenging for interactions between science, policy and publics over the next 10 years in the UK”. In the process of scoring, this group also suggested areas where some of the issues raised could be amalgamated to deal with overlap and duplication. The scores were collated and the medians calculated to provide the basic material for the Workshop.

Finally, people drawn from both the previous groups and chosen to provide a wide range of expertise and a broad coverage of both the issues, the organisations and the sectors involved were invited to participate in the Workshop; we invited 131 in the expectation of getting between 50 and 60 positive responses (previously found to be a suitable number for this type of exercise [1]), 55 of whom accepted and are listed as authors of the paper.

Of those originally invited, 47 (36%) came from Government Departments and the wider public sector, 30 (23%) from public policy consultancies, NGOs, Industry and professional science journalism, and 53 (41%) from the science community, including academia, research councils (and their institutes) and learned societies. These groupings overlap in many ways; nearly 40% of the public sector group are active scientists and several in academia have experience in public policy. The same is true for those who participated (public sector 18 (33%) of whom 9 are active scientists; Consultancies, NGOs and industry 6 (11%) of whom 4 are active scientists, and science community 31 (56%)). With respect to coverage of the issues raised in the long list of questions and the spread of expertise required, the participants, being, largely, at experienced middle to senior management levels, brought with them not only their own specialist knowledge but also a broad awareness of wider S&T developments.

Initially, four sub-groups of participants worked in parallel sessions to discuss sub-sets of the 100 issues (for example, all the issues related to food or health or security). Each sub-group dealt with one of these subsets of issues at a time and, initially considering the previous scoring, (especially in removing low scoring issues), identified, elaborated upon and defined the three top issues and one runner up in each subset. For each of these four issues, the sub-groups then identified the five main science and technology challenges that might result from or affect their emergence.

There followed a final plenary session, at which all participated in a process of discussion for and against the inclusion or exclusion of issues from the priority list. Voting on inclusion in the final list was carried out according to the same criteria of which issues were most likely to be challenging for interactions between science, policy and publics over the next 10 years in the UK. The process resulted in a final list of 30 agreed priority issues.

These 30 remaining issues were once again broadly grouped into subject areas (e.g. environment, IT) and participants re-divided into groups each focused on one subject area. Their task was to examine the consequences of each of the issues selected (i.e. to review the policy issues and S&T factors affecting them); these were then written up in a standard format of explanatory text, list of science and technology challenges, and references.

Following the initial drafting, all participants had the opportunity to edit the paper electronically; this was an extensive process, over a number of months, of iterative redrafting, which enabled challenge to the framing of the issues and challenges, and to the conclusions.

We did not obtain ethics approval for this exercise, as it was agreed from the outset that all of those participating in the Workshop in the voting and selection of the issues were to become authors of the resulting paper. However, all the initially submitted questions were treated anonymously; and it was agreed that publication should be in an open-access journal, if possible, in order facilitate general accessibility for those in policy communities.

## Results

We present the results of our discussions in the form of a title for each of the 30 emerging policy issues identified, supported by a brief summary of the current state of knowledge with a set of conclusions about the emerging science and technology challenges for policy makers.

### 1. Novel Bespoke Models of Consumption and Production

Additive manufacturing techniques offer the promise of cheap, local, low volume production. One of the expectations of this technology is that it could allow consumers to design, customise and manufacture personalised items, creating an industry for bespoke manufacturing and open source design [24]. While 3 D printers could potentially follow the PC in moving from industry to the home [25], there is a real opportunity for businesses to create local manufacturing services to support this new model of consumption. The technology also offers enhancement of consumer choice through making items available in the long tail [26], [27] of low demand products at low cost to the consumer compared to standard manufacturing. Additive manufacturing could offer sustainability benefits by reducing energy used in distribution by shrinking distribution chains and limiting waste from warehousing and overproduction. It could also avoid some of the waste inherent in subtractive manufacturing processes. However, the powders and polymers used in additive processes are often hazardous, come with embedded energy and require their own distribution networks, and users may print unneeded items on a whim, creating a new waste stream. Concerns arise about controlling the standard of items manufactured from blueprints available on the web, and where liability rests in the case of product failure. Controlling intellectual property presents a challenge when items can easily be rendered in CAD blueprints from a picture; as will controlling the manufacture of illegal or controlled items or their parts [28].

**Science and technology challenges:**

- Assessing the net impact of bespoke manufacturing on the economy, employment and length of the supply chains
- Conceiving new models for resource use efficiency, including the reduction of waste after use and during production, and minimisation of embedded energy and raw materials
- Developing appropriate Intellectual Property Rights and standards
- Understanding possible changes in digital retail and manufacturing, including issues such as liability and safety
- Understanding localised mass customisation and the actual effect on business models

### 2. Innovation: The Role of Government

A key function of government is correcting market failures, with respect to which, an important activity is to generate an environment that encourages innovation to the benefit of businesses, consumers and society. Government needs to work in concert with both industry and academia to stimulate, support and maintain a framework for innovation, including the innovations sought to deliver public as well as private goods [29], [30]. The exploitation of public data and greater dissemination of the results of publicly funded research also have an important role to play. In particular, while private sector R&D can drive incremental and sometimes radical innovation, Government investment in research is essential to support transformational innovation [31]. Silicon Valley’s apparently joined-up innovation ecosystem, involving clusters of related industries, seems to exemplify a successful model, but the UK is not California and we need our people to understand and support the conditions for successful innovation here. Governments cannot create new clusters [32], but can encourage new collaboration and remove the obstacles that inhibit clusters from growing.

**Science and technology challenges:**

- Communicating the benefits and risks of innovations, to achieve wide understanding and informed choice
- Understanding, developing and refining national systems of innovation, including a framework and standards for promoting innovation and Government procurement
- Ensuring innovation systems support societal as well as private objectives and deliver on issues such as ICT and social inclusion, the protection of ecosystem services, carbon reduction and antibiotic resistance
- Ensuring innovation systems accelerate radical as well as incremental innovation
- Developing a greater understanding of what delivers transformational change

### 3. Energy Resilience and Low Carbon Industry

The electricity regulator Ofgem estimates that £200 billion of investment in energy infrastructure will be required over the coming decade, to decarbonise the electricity grid and transform energy use [33]. Historically, the UK has invested in large-scale centralised electricity supply, with fewer than sixty large power stations. This is however changing. A large-scale supply will still be needed, which is likely to include offshore wind and nuclear power. However, renewable energy is often at smaller scales and more distributed (e.g. building-integrated solar photovoltaics and renewable heat grids) and energy networks will increasingly mesh with IT networks, through smart metering and other information technology [34]. Web-based crowd-funding platforms could transform energy investment models, opening up the energy market to new entrants, including co-operatives and community schemes (e.g. Abundance Generation https://www.abundancegeneration.com/and Trillion Fund http://www.trillionfund.com). Meeting carbon targets will require a fundamental look at how we use energy too, with transport networks and land-use planning influenced by the need to make smarter use of energy [35]. Encouraging this shift requires changing government policy, including better incentives for innovation in microgeneration networks as well as generation technologies; a more strategic land-use planning system for energy infrastructure; a move beyond energy efficiency to incentivise demand management measures in transport, housing and industry; and the encouragement of new entrants into energy investment, including local authorities, co-operatives and the ICT sector.

**Science and technology challenges:**

- Incentivising low carbon technologies and production
- Presenting the case for planning permission/consent for energy infrastructure
- Promoting interdependency in decision making with other areas, e.g. transport, housing
- Incentivising investment in the demonstration of technologies
- Accommodating both centralised energy systems and distributed generation (see also next section)

### 4. Policies for Whole Energy Systems

As an era of dependence on fossil fuels gives way to emerging efforts to transition to a low carbon economy [36] (also see previous section), energy systems are becoming more distributed and interconnected [37] and such trends stand to increase substantially over the next few decades. Centralised energy supply from large power plants is increasingly accompanied by smaller scale production (e.g. renewables, micro-generation) and storage. Energy demand reduction and efficiency measures need to be taken more seriously, giving a wider range of actors (including consumers and households) and technologies (e.g. smart devices) a more active role [38]. There must also be more focus on demand shifting to allow better use of the dynamic supply created by renewable energy sources [39]. Energy security, once a predominately national concern, becomes one of interdependencies between nation states. Under these conditions a compartmentalised policy approach – that focuses on specific energy technologies, sectors, and parts of the system in isolation – becomes outmoded. Secure, affordable and environmentally sustainable energy futures require a systems approach to energy policy, and a policy for the whole UK energy system covering generation, transmission, distribution and use. This will involve integrated and joined-up policies that account for interconnections across the system and scales of decision-making. This in turn depends on developing whole systems energy science that understands system-wide interconnections and interdependencies through linking physical, engineering and social scientific analyses [40].

**Science and technology challenges:**

- Developing capabilities in interdisciplinary whole systems energy science, including new methods, approaches and analytical tools
- Understanding the implications of a whole systems approach for structuring, governing, regulating and managing the energy system and low carbon transitions
- Devising mechanisms to facilitate integrated grid management systems and utilise SMART technologies
- Determining the economic dimensions and market implications of a more distributed energy system
- Developing and harnessing robust evidence of the social and political dynamics of energy transitions in tackling difficult decisions around energy systems

### 5. Energy and Transport Infrastructure for Changing Work and Living Patterns

Work and living patterns are changing due to increased usage of information and communications technologies (ICT). Individuals now have more flexibility over where they live and work. These changing patterns, and how they impact on energy and transport infrastructure, need to be better understood to inform policy decisions. Benefits of these changes range from reducing peak demand in capacity-stricken transport systems, reducing associated negative environmental impacts [41], [42], more efficient business organisation and improved work-life balance for workers. However, these shifting patterns could significantly change the *expected demand* for services provided by energy, transportation and also ICT infrastructure. This is an issue for the UK as it has many infrastructure challenges to tackle in the coming decades, particularly with regard to climate change [43]. Emissions may shift from transport to home energy demand or may re-emerge in other parts of the system. More robust quantification of these changing patterns are needed. The ‘behavioural turn’ in policy making [44], suggests that policy responses will be more effective if they incorporate understanding of the drivers that influence behavioural change [45], coupled with understanding of the *long-term evolution* of energy, transport and ICT systems, and their changing relationships to the environment, the economy and society.

**Science and technology challenges:**

- Measuring, understanding and quantifying infrastructure demand changes on energy, transport and ICT
- Identifying opportunities that could arise from changes in infrastructure demand, such as in ICT reducing transportation demand
- Understanding the interactions of the energy, transport, ICT and social systems and how they influence the evolution of the systems
- Understanding how changes in energy, transport and ICT systems affect total emissions
- Identifying drivers that will influence long-term behaviour change, utilising them for societal advantage

### 6. Feeding a Larger and Wealthier Global Population Sustainably and Equitably

Today nearly a billion people suffer from calorie hunger and a further billion are malnourished [46]. Food demand will increase dramatically driven both by growing population but also by higher average wealth leading to demands for more resource intensive diets. Possibly 60–70% more food will be needed by mid-century to avoid politically destabilising food price increases [46]. Addressing famine and malnutrition are ethical priorities as well as essential for politico-economic stability. Food production is also threatened by greater competition for water, land, energy and other inputs, and well as the effects of climate change. Agriculture is already a major agent of environmental degradation, undermining our future capacity to feed the global population, as well as a significant driver of land use change, nitrate pollution and greenhouse gas emissions. There is an urgent need to make food production more environmentally sustainable; enhancing and not degrading natural capital. The magnitude of the challenges ahead strongly suggests that action is required simultaneously on all parts of the food system – to produce more food, consume less resource-intensive food types, reduce waste and improve the governance and efficiency of the food system [47]. Though bringing more land into agriculture would increase food production, the advantages are outweighed by its major environmental consequences (including greenhouse gas emissions and loss of biodiversity) [48]. Sustainably increasing productivity (sustainable intensification) is thus a key response [49].

**Science and technology challenges:**

- Reducing waste and improving efficiency in storage and distribution
- Producing more food from the same area of land with fewer negative environmental effects
- Increasing the efficiency with which water, energy, fertiliser and other agricultural inputs are used whilst also increasing agricultural outputs
- Addressing the food needs of “mega-cities”, especially in the tropics
- How choice of diets could be influenced towards those with less environmental (and health) impact
- Creating governance frameworks for the global food system to promote food security

### 7. Sudden Environmental Change

There are growing concerns about the accelerating rate of environmental change as a result of continuing population growth and resource consumption [50] Climate change represents an unprecedented and sudden change relative to climate variability over the last 12000 years, posing considerable challenges for risk management and public action with a consequent need for transformational adaptation [51]. How we rapidly and deliberately transform systems and society in order to avoid the long-term negative consequences of sudden environmental change, however, remains a considerable challenge [52]. Changes in the climate system are occurring at the same time as other environmental changes, such as biodiversity loss and ocean acidification. A range of planetary boundaries has therefore been proposed [53] that define the ‘safe operating space’ for humanity with respect to the Earth system. There is, in addition, an increasing awareness that once a critical point is reached, positive feedbacks in the system may propel change towards an alternative state [54]. A number of such critical points, sometimes referred to as tipping points, have been identified, including the loss of Arctic summer sea-ice, reorganisation of the Atlantic thermohaline circulation and dieback of the Amazon forest. The risks and economic consequences (and potential benefits) of such tipping points remain difficult to assess [55].

**Science and technology challenges:**

- Identifying when we should respond to the risk or opportunity of extreme environmental changes
- Developing an appropriate policy approach for low probability but high impact events
- Learning from past high impact singular events and examine whether they were predicted by models in use at the time
- Understanding public perception of risks and how these can be taken into account in decision-making
- Encouraging researchers to accept more openly the limitations of their research and models, and enhancing capability in communicating uncertainty, ambiguity, complexity and ignorance

### 8. Climate Change Adaptation

Societies adapt pragmatically to variations in current weather. But climate change presents adaptation challenges of a different order as extreme events become more frequent and widespread. Building resilience to climate change requires actions across every sector – from agriculture to health, from business to infrastructure – and the interactions between them. Actions are likely to have a net cost, compounded by the fact that there is no defined adaptation end point. This creates a significant institutional and societal challenge, locally and nationally [56], [57] with an outstanding need to build appropriate governance systems and supporting analysis at different scales. The UK Climate Projections [58] provide readily accessible probabilistic projections for future climate. But the first Climate Change Risk Assessment [59] illustrates the problems of dealing with endemic uncertainty and complexity not least in terms of the interrelationship between on-going environmental, economic, and societal changes and climate change; see also the National Adaptation Programme [60]. There remains an urgent need for institutions to build adaptive capacity into every element of their responsibilities with a cross-sectoral approach to policymaking and implementation [56]. Effective institutional responses will build flexibility into decisions such that future as yet unknown climate outcomes can be responded to in a timely and cost-effective manner while maintaining a positive contribution to ecosystem services.

**Science and technology challenges:**

- Identifying governance system options that could enable effective local and national adaptation responses
- Identifying and defining climate adaptation priorities, e.g. temperature extremes or longer-term temperature changes
- Analysing the full range of the local impacts of climate hazards and their interaction across different sectors to support planning at the local level
- Understanding the potential consequences of series of different climate related hazards
- Applying broader and more sophisticated systems thinking to analysis and policy-making that reflect the complex interactions between and within environmental, economic and social systems

### 9. Climate Geo-engineering and Climate Change

Geo engineering [61] refers to deliberate, large-scale intervention in the global natural systems that determine climatic patterns. Concerns about whether we can mitigate climate change through emission reduction sufficiently fast, or adapt to temperature changes in the higher of predicted levels, have led to consideration of such technologies as an alternative approach to mitigation of climate change effects. Currently identified possible interventions [61] include direct atmospheric modification (such as increasing cloud formation, thereby raising planetary albedo), atmospheric/oceanic (ocean fertilisation) or atmospheric/land surface (surface albedo modification) interactions, as well as manipulation of the earth’s incoming solar irradiance in outer space. A subset of climate engineering is the so-called Negative Emissions Technologies [61], [62]; NETs are intended to remove significant amounts of radiative forcing gases (usually CO_2_) from the atmosphere and include such diverse methodologies as ocean fertilisation and the large-scale pyrolysis of vegetable matter, with CO_2_ capture from the flue gases, followed by its geological sequestration and the burial of the remaining char in soils, which can lock up its contained carbon for many years. All geo-engineering technologies need to be assessed in terms of energy use, economic costs, social acceptability and environmental consequences; they may exacerbate climate risks rather than reducing them if not carefully managed [63]. In consequence, a set of principles for undertaking research into geoengineering options [64] was discussed and endorsed by the House of Commons in a recent report [65] calling for

1: Geoengineering to be regulated as a public good

2: Public participation in geoengineering decision-making

3: Disclosure of geoengineering research and open publication of results

4: Independent assessment of impacts

5: Governance before deployment

Making these principles operable will require research and testing.

**Science and technology challenges:**

- Identifying the potential impacts and unintended consequences of the various technologies
- Identifying and addressing the implications for international agreements
- Assessing the scalability, rollout potential, effectiveness and reversibility issues associated with each of the technologies
- Identifying the social impact and acceptability of the price of carbon that is required to ensure that these approaches are cost effective
- Dual use dilemma and other risks, but also whether these technologies could provide opportunities for other public benefits

### 10. Integrated (Multi-functional) Land-use Planning

Robust land use policies and delivery mechanisms are vital to the economy, for food, commodity and energy production and for the use of land for infrastructure, housing, tourism and recreation. UK approaches have traditionally focussed on single purposes, often to enhance delivery of one ecosystem service, such as food provision, to the detriment of others [66]. From the late 1940s, the UK focussed on maximising production, but while productivity increased other ecosystem services declined, particularly biodiversity and the quality of air, water and soil [67]. Population increase, climate change and economic growth, taken with the finite nature of land and natural resources, present a growing concerns for policy makers as to the continued delivery of a range of ecosystem services [68]. The food, energy, environment “trilemma” for land use, if poorly handled, may lead to further ecosystem degradation, increasing energy consumption and missed opportunities (e.g. to introduce lower carbon feedstocks, optimise food production at less ecosystem detriment or incorporate biofuels into land allocation strategies to reduce fossil fuel use [69]). ‘Multi-functional’ approaches to land use propose balancing competing demands across wide areas (landscape scale) to ensure multiple ecosystem services are delivered. Emerging new methodologies to measure and value benefits provided by environmental assets could improve policy decisions about species, habitat and ecosystem conservation or conversion [70]. Such evaluations bring social & personal, as well as economic, values into focus, necessitating new approaches to engaging the public about their environment and in how it is used.

**Science and technology challenges:**

- Assessing the validity and usability of different approaches to valuing ecosystem services
- Developing scenarios for addressing the land use ‘trilemma’
- Evaluating approaches to trading off and optimising between ecosystem services in resource allocation decisions
- Improving the ability to analyse risk and opportunity in resource and service allocation decisions
- Developing and testing methods to support resource allocation conflict resolution

### 11. Novel Substances in the Environment

As technologies develop there is a need to effectively identify and assess previously unidentified risks [71]. Communication and regulatory challenges are complicated by incomplete evidence bases and the need to balance opportunity and caution when the impact of, or exposure to, a substance is unknown [72], [73]. Development should be socially responsible and use appropriate regulatory tools [74]. Governments rely on methods (e.g. PESTLE analysis, considering the Political, Environmental, Societal, Technological, Legal and Economic impacts) to support responsible decision-making [75], but in industry less encompassing approaches are used. Technological advances mean that the impacts of new substances (or technologies) may not be characterised until they are in widespread use [76], further complicating regulatory decisions, e.g. nanoparticles used to scavenge soil contaminants are now known to have additional effects. Normally assessors use a dose-response to identify hazards, but this may be misleading without understanding how the target organism processes the substance (e.g. accumulation, metabolism or excretion). Researchers may assume a linear or threshold response and design experiments accordingly, thereby missing non-standard (e.g. U shaped) responses [77]. Some adverse effects may not be identified by standard test regimes if they occur at low doses but may be of concern where receptors are exposed to the substance by many routes or when a number of substances have similar affects (e.g. endocrine disrupting chemicals [78]). Such limitations affect the appropriateness of adopting governance principles, such as ‘substantive equivalence’ or ‘precautionary principle’.

**Science and technology challenges:**

- Establishing timely risk identification and effective management responses to emerging novel substances, also determining the acceptable levels of risk
- Developing and promoting a balanced, adaptive, responsible approach to the governance of innovation which considers both risks and benefits of technological advances
- Identifying opportunities for the exploitation of novel substances, such as the use of scavenging nanoparticles in remediation
- Understanding and measure dose response curves in analysis, and deal with the consequences when things go wrong
- Explaining and ensuring the context specific, appropriate application of risk frameworks

### 12. Meeting Long-term Skills Requirements

Concerns about meeting long-term skills requirements have been persistent; underpinned, first, by difficulties in predicting and anticipating demand, and second, by challenges to meeting demand through UK-based education and training as well as absorption of skills available from other countries. In the first case, predicting and anticipating demand for skills is beset by inherent uncertainties: it is difficult to know what skills sets will be needed by society in future production or in the provision of future services, and how these skills sets might be changed (or even eliminated) by advances in technology. In the second case, meeting demand for skills through education, training and absorption is no less difficult, requiring greater understanding about and enhancement of learning, skills development, and national absorptive capacity. Alongside cultivating and maintaining high levels of scientific literacy at national level, individuals should also be supported in ‘learning to learn’ [79] (i.e. learning through applying knowledge and skills flexibly in a variety of roles, and treating learning as a life-long endeavour) and be empowered to make proactive careers decisions. This is likely to require even closer collaboration between government, industry and the education sector, working through formal and informal channels of education. In relation to the last, more strategic research and systematic evaluation of initiatives are required to help build on knowledge that is being generated from experience [80].

**Science and technology challenges:**

- Improving methods for anticipating demand,
- Developing enabling frameworks to help individuals to make proactive careers decisions coupled with improving individual capacity for learning and acquisition of skills
- Understanding and enhancing national absorptive capacity for skills from elsewhere
- Maintaining long-term scientific literacy (at national and individual level)
- Providing a strategic evaluation of interventions

### 13. New Means of Delivering Learning

The delivery of education using the Internet promises to bring learning opportunities to more people than ever before, potentially drawing new audiences from across the world, whilst also enabling learners to have greater control over when and how they learn. Massive open online courses (MOOCs), in particular, are growing rapidly in popularity. This expansion in e-education, however, is not without challenges. On a practical level, sustainable business models for commercialising MOOCs, encompassing systems for verification of credentials, assessment and certification, have yet to be developed [81]. Whilst it is hoped that specially-prepared lectures and online content may drive improvements in teaching and ensure a high quality learning experience for more learners, issues around quality assurance and ways of accessing reliably-verified information have yet to be resolved. Research is also needed to help understand the potential of e-education within both formal and informal education contexts, and how this can be exploited to enhance learning. As yet, development of good practice is unsystematic. Little is known about the relative efficacy of widespread e-education, particularly as it relates to the social engagement of learners, which is thought to be valuable in its own right as well as being an important factor underpinning motivation and performance [82], [83]. Widespread access itself is also predicated on the deployment of super-high-speed broadband. The challenge of providing this necessary technological infrastructure poses questions for democratic access to information and learning.

**Science and technology challenges:**

- Providing reliable verification of information (quality assurance) and access to reliably-verified information
- Developing systems for verification of credentials, assessment and certification, particularly for distance learning
- Developing sustainable business models for MOOCs and other novel approaches
- Presenting scientific information in ways that are accessible and intelligible to non-scientific audiences
- Understanding social engagement through educational practises and how its benefits may be captured in e-learning contexts

### 14. Assessing the State of the Nation

Whilst policy-makers’ desire to assess the overall “state” of the nation is not in itself novel, many workshop attendees felt, in light of a persistent lack of economic growth, that the coming years would witness a renewed imperative to find more comprehensive measures of ‘success’ than simple Gross Domestic Product (GDP). According to a 2012 House of Commons Public Administration Select Committee Report [84], the incumbent UK Government has itself identified six ‘strategic aims’ as crucial to the advancement of the national interest: (1) “a free and democratic society, properly protected from its enemies”; (2) “a strong, sustainable and growing economy”; (3) “a healthy, active, secure, socially cohesive, socially mobile, socially responsible and well-educated population”; (4) “a fair deal for those who are poor or vulnerable”; (5) “a vibrant culture”; and (6) “a beautiful and sustainable built and natural environment”. This policy issue is therefore concerned with finding robust and appropriate measures (such as of wellbeing and happiness; see [85], [86]) of the extent to which such strategic aims are being met [see also entry 25 below – on the use of happy life expectancy as a criterion for resource allocation decisions by government]. At a more fundamental level however, it also pertains to the question of what exactly Government should be seeking to measure in the first place. Recent efforts in this direction include the UK National Ecosystem Assessment [67] and the on-going work of the Natural Capital Committee, for example their The State of Natural Capital report [70] to the Chancellor of the Exchequer.

**Science and technology challenges:**

- Comparing the relative strengths and weaknesses of different measures of success, productivity, happiness, wellbeing and other desirable attributes in society today
- Criteria for choosing appropriate and robust metric(s) for the measurement of national success that go beyond simple GDP
- Developing research tools and methodologies that are able to capture this/these metrics accurately
- Allocating fiscal and other resources between and within Government departments in ways that enhance the “state of the nation”
- Developing evaluative tools that will enable policy-makers to respond to data concerning the state of the nation promptly and effectively

### 15. System-level Vulnerabilities and Increasing Complexity

Policy has to deal with numerous multi-faceted complex issues where evidence is often fragmented, highly uncertain and comes from a range of sources [87], [88]. These situations are also notable by the many highly interrelated aspects and contested issues that arise and where deep-seated conflicts around purposes, goals and values are common. Complexity and interdependence give rise to often unpredictable ‘emergent’ outcomes, belying the single-point forecasting approaches traditionally used in developing and implementing policy and creating vulnerabilities for decision-making [89]–[91]. Yet, in the face of such profound and ubiquitous complexity and interdependency, policy still seeks to promote positive outcomes for society, the economy and the environment [87]. While systems-theory and complexity science are developing fields [88], [92], they are not widespread in policy or practice, requiring tailor-made approaches [89], [93]. Exploratory and experimental attitudes and tool-kits are required that allow us to adapt as we learn from practice and experience across many domains [90], [92]. The application of science to policy requires integration of (a) Different sources and forms of knowledge (natural science, social science and humanities, practice-based knowledge, lay knowledge, etc.) [87], [89], [93]; (b) a range of processes for acquiring, making sense of and using this evidence [91], [92]; and (c) a range of different perspectives and approaches (e.g. [94]).

**Science and technology challenges:**

- Developing applications of systems thinking and complexity theory for policy making
- Applying systems approaches across disciplines
- Identifying conditions under which systems may be vulnerable to failure and understanding the resilience of systems
- Understanding how to allocate resources to high impact but low probability events
- Understanding non-linear systems within a policy context (e.g. the electronically connected world)

### 16. National Infrastructure in a Localised and Interconnected World

Historically, the implementation of each individual infrastructure project has been treated as an isolated technical challenge, with only sufficient integration to meet the project aims. The Council for Science and Technology [95], however, recognise that, in reality, national infrastructure is better considered as a network of networks. This is reflected in the National Infrastructure Plan of 2011 and subsequent updates [43], [96] which noted that the UK’s approach to infrastructure had thus far been fragmented, adding that “opportunities to maximise infrastructure’s potential as systems of networks have not been exploited”. These infrastructure interdependencies can be physical, digital, locational or organisational and such complex systems can suffer from common mode failures or precipitate failures that ‘cascade’ from one sector to another. Planning and managing these interdependencies can increase system resilience and save engineering costs, but can concentrate risks. Reliance on digital and organisational interdependencies for the continued safe delivery of services by national infrastructure networks introduces a number of common mode failure possibilities and new vulnerabilities to attack. A balance must be struck between security and the higher levels of efficiency that smart systems allow. The emergent behaviour of complex, interconnected and interdependent systems (especially open complex adaptive systems) cannot be adequately modelled or always predicted following perturbations to any component of the system. Planning, management and policy responses therefore need to be flexible and adaptive.

**Science and technology challenges:**

- Enabling efficient and effective public engagement and access to information
- Understanding incentivisation, including the science of offsetting
- Improving contextualisation (science input) to inform high level decision making
- Improving understanding of how to identify at an early stage and manage emergent properties of complex systems
- Enhancing government understanding and evaluation of the validity and value of different forms of knowledge

### 17. Public Sector Capacity to be an Intelligent Customer of Scientific Advice

This is a different kind of issue from others addressed in this paper, but its importance comes from the way in which it addresses the effectiveness with which other issues are handled. It is also driven by the observed difficulty in having open and objective debates on the evidence surrounding socially and ethically sensitive issues, such as recreational drugs [97]. It has never been the case that all scientific advice and evidence is generated within Government. In the current context, with the current Civil Service Reform plans, and the move to open policy making [98], it is even more imperative that the public sector has the capacity and expertise to be a commissioner and customer for scientific advice, and to ensure that the scientific aspects of an issue are weighed in the balance with others. Further, the open policy making agenda will put an even higher premium on the ability to moderate an open discussion on difficult issues, where the pressure from the media is sometimes liable to drive a more knee-jerk approach. With respect to the use of scientific advice in policy making, and the development of the Science and Engineering profession in Government [99], important issues include the challenge of retaining knowledge when officials move post, and its impact on understanding; the importance of an active and reliable network of contacts in the scientific world, when an urgent issue (such as foot and mouth disease) needs to be handled; and handling the research commissioning process. Similarly, it is critical to build capability in scientists, and policy-makers (and wider constituencies) to recognise, characterise, take account of and communicate inherent uncertainties, ambiguities, complexities and knowledge gaps inherent in science and evidence.

**Science and technology challenges:**

- Re-evaluating the effectiveness of current structures for obtaining advice and for investing in future evidence provision through research, including whether they are understood, sufficient, and appropriate
- Developing the foresight to identify research needs of government departments (as opposed to the Research Councils & Higher Education sectors)
- Identifying an appropriate range of policy evaluation approaches and methodologies (including working outside of policy and discipline silos)
- Creating capacity for more creative thinking about ‘wicked problems’
- Developing methods for assessing the value and validity of, and for valorising, non-scientific evidence in policy making

### 18. Using Public Engagement to Improve Government Decision-making

Within the UK Civil Service Reform agenda [98], government is trying to develop better ways of bringing public opinion and public views into the early stages of policy development [100]. This requires a shift in the culture to empower civil servants to put forward genuine questions at an early stage for resolution to the wider public, rather than simply offering pre-prepared solutions for validation by the more usual 6–12 week public consultation process. Civil Service Reform has signalled [101] the need to change the traditional policymaking cycle in order to encompass public/stakeholder participation earlier, and to foster a greater understanding of how to frame questions that will encourage input that is relevant and timely, and therefore useable. This has highlighted the need for improved capability among policymakers in analysing and interpreting the public responses. Automated analysis tools are low in resource terms but not always deployed effectively. New dashboards are currently being rolled out in a number of Departments to monitor social intelligence, but the value of the data is often not maximised to support decision-making. Currently it is used more as a horizon-scanning tool to predict the likely reception of Departmental policies and so to inform their communications plans. There is a gap in political and social science analytical skills at the cutting edge of public engagement, most especially when social media channels are in use. This has created a risk that the dialogue and data capture methods now available are outstripping the analytical capability.

**Science and technology challenges:**

- Understanding how to interpret and have confidence in evidence and outcomes from public engagement
- Determining when (and when not) to engage the public
- Using crowdsourcing and social media technologies to gather public opinion and generate public discussion
- Understanding and developing responses to the internal mechanisms of policy-making that make action on social intelligence 'difficult' for policy-makers and politicians, through applying political science to develop and trial new approaches
- Developing new methodologies for government to monitor social intelligence

### 19. Democracy in the Digital Age

Technology is changing the way that all engagement between institutions and citizens is undertaken [102]. The first generation of digital engagement often just replicated paper forms on a website, but second-generation models are more social, more flexible and more conversational [103]. The UK Government’s Open Policymaking programme [98] has recognised this and is seeking to change practice in Whitehall and beyond. A digital approach can support good policy development in two ways: first, online engagement around policies involving science and technology will increase the ability of the public to participate in democratic discussion. Secondly, where specific exercises are planned, digital methods can expand the footprint, involving more people and broadening the conversation. However, digital routes can quickly spread misinformation that distorts or oversimplifies information, thereby undermining related policy debate. Similarly, digital media can exacerbate the problem of over-simplifying complex technological points, and can also be used to present a baseline of opinion rather than knowledge. Looking to the future, many see digital technology as much more than an alternative delivery mechanism; it is a culture and an approach. Digital media is seen as increasingly likely to dominate engagement in science policy, perhaps even becoming the primary portal for debate rather than an electronic reflection of offline activities.

**Science and technology challenges:**

- Identifying the issues involved in balancing the individual’s right to privacy with the use of data (public or private) for public good and approaches to resolution
- Developing criteria for balancing participation (access issue and control issue) and inclusivity (e.g. with respect to interest groups vs. other stakeholders)
- Identifying the hidden implications of search algorithms and filtering of knowledge (the geography of internet control) and finding approaches to manage them transparently
- Understanding perceptions and intentions of “transparency” and “open decision making” and how to achieve them
- Providing sufficient but not excess access to reliable information that meets the needs of individuals and social entities

### 20. Managing Extreme Events: Public and Private Sector Roles

Individual national governments no longer have exclusive ownership of the infrastructure and delivery mechanisms that previously provided resilience in times of environmental or social emergencies [104]. The water, food, power and transport systems are all owned by private, often global, entities that may not always share national or domestic community imperatives. The solutions to food shortages, flooding, energy outages are traditionally dependent on the co-ordinated delivery of support services and emergency plans. Since these are now effectively outsourced to private sector companies - who may need to respond to market requirements rather than urgent local needs – how can governments continue to provide the necessary duty of care to its citizens? Incidents that require manpower are still relatively simple to resolve using employees within the public sector, e.g. the army are deployed to support flood evacuations; Local Government employees can man the farm quarantine areas during animal outbreaks. Emergencies whose solution is expected to involve distribution of now privately owned assets or service delivery (e.g. energy, water, fuel) require a different form of contingency planning [105]. If the emergency is one that impacts the priority customers of the asset owners, then the two agendas are aligned. The question arises when – in the event of a collective failure, perhaps linked to an extreme weather event (sunspots, flooding and volcanic ash) – the global company has to select the countries to which it prioritises resources.

**Science and technology challenges:**

- Identifying social, economic and technical barriers to engagement and collaboration between organisations in the response to extreme events, especially public and private organisations, and ways to overcome them
- Identifying the distribution of collective and individual responsibilities and liabilities for emergency response and contingency planning between public and private organisations and means to share them equitably
- Assessing whether insurers could provide mechanisms for cost and benefit sharing, especially in relation to low incidence high damage emergencies
- Evaluate the implications of cost and benefit distribution for investment in infrastructure provision
- Investigate alternative business models for private sector companies which could enable them to address their responsibilities for planning for and responding to disaster situations

### 21. Emerging Diseases

The threats from emerging diseases across the plant and animal kingdoms are an ever-present worry with increasing human populations, increasing intensity of crop and livestock production, pressures on scarce resources, climate change and the international mobility of man, animals, plants and organic materials [106]. Just as threats to human health may come from zoonotic infections or from infectious agents that jump the species’ barriers, so might threats to the environment come from transmission of disease from domesticated to wild populations [107]. The threat is on a global scale; greater mobility, combined with changes in environment, threatens each nation. We need to understand and to anticipate the effect of environmental changes that have seen the recent appearances in the UK for example of the Schmallenberg virus or the emergence and relatively rapid spread of *Chalara fraxinea* as the causal agent of dieback of Ash trees in Europe [108]). We need to understand the complexities and dependencies of systems (such as agriculture and trade), looking for commonalities as well as the specificities of approaches to complex problems including better computer modelling, increased understanding of the fundamental principles of infection, transmission and species barriers, genetic and environmental factors affecting susceptibility and triggers for emergence or resurgence of disease threats. We need to make use of novel research tools both for surveillance [109], imparting information and also crowdsourcing afforded by the globalisation of access to electronic media [108], understanding not only the challenges, but also the opportunities this brings.

**Science and technology challenges:**

- Identifying and prioritising disease threats in plants and animals (including humans) and the effects they have on the different ecosystems (including services)
- Improve institutional risk analysis & management systems to enable better integration of early-warnings into priority setting and resource allocation within business-planning
- Understanding the mechanisms that enable diseases to jump the species barrier and how they emerge in populations
- Developing better models for understanding disease spread and control, and feed that through to the decision making process in a timely fashion
- Determining the role of social media in identifying disease outbreaks and influencing public action and perception

### 22. Antimicrobial Resistance and Infectious Diseases

The UK Chief Medical Officer has identified antimicrobial resistance as one of the greatest threats to modern health [110], a view shared by the World Health Assembly [111]. Antimicrobial resistance in pathogens that affect human health is an unsurprising evolutionary consequence of the prevalence of antimicrobials in human and animal populations. The rate of its development is a function of how widely they are used and to what degree in any domain where dangerous pathogens are present. The science and technology challenges related to this issue, therefore, are to be understood in the broader context of how human societies respond to any infectious disease, since pathogens will always sit in a dynamic and evolving relationship to the species they infect. Antimicrobial resistance represents a distinct policy challenge [112] only because of our failure to take account of evolutionary processes in the conduct of medical practice and the wider use of antimicrobials. This is exacerbated by trade and travel affecting the global circulation of resistant organisms. A two pronged approach is required that addresses not only the scientific, technical and industrial agenda for the prevention of infection, promotion of immunity and facilitation of diagnosis and cure, but also political, social and economic issues [113] that are fundamental to the translation of scientific discoveries into effective and sustainable practices

**Science and technology challenges:**

- Resolving the tension between public health requirements and personal preference
- Identifying and promote measures that prevent people from getting infectious diseases, for example avoidance and vaccinations
- Monitoring and understanding the emergence of novel infectious agents
- Identifying and promote behavioural change (misuse of antimicrobiotics) and improved diagnostics (input of genomics and proteomics) that will reduce infectious diseases
- Identifying methods to incentivise the development of novel therapeutics

### 23. Effective Health Systems within Finite Budgets

With an ageing population and the increase in chronic conditions like diabetes, demand for healthcare looks certain to increase [114]–[116]. Patients’ expectations of the quality of service and support that they receive will continue to be high. Scientific developments in pharmaceuticals, medical devices, imaging technologies, diagnostics and improved ICT systems will generate opportunities for improved care that are important for patients, and are areas where the UK life science community is looking to advance research and commercialisation of new products [117]. However, fiscal constraints on government spending [118] mean that budget limitations will continue to constrain what can be offered to patients via the NHS. Other types of innovation to reduce utilisation of services through better self-care or home care may generate opportunities for efficiency gains [119]. Adoption of low-cost technologies and approaches piloted around the world may also create new ways to strip out costs from current modes of service delivery. Understanding the emerging technical opportunities for healthcare innovation and the social factors that will drive future demand and utilisation of health services will be essential to allow the UK to meet its long-term policy objectives of improving health outcomes and generating a world-leading life sciences sector within a context of fiscal probity.

**Science and technology challenges:**

- Developing better tools for self-management for (chronic) diseases
- Improving the balance between prevention and treatment
- Understanding and managing demands for healthcare from the public
- Developing technologies that have high impact but may have low market value or relate to unfashionable areas of research
- Developing or borrowing useful low-tech solutions from around the world

### 24. Demographic Change

One of the most powerful levers affecting future policy is demography. It affects the provision of resources, such as food and water, services, such as health care and pensions, and has profound effects on the economy. However, the importance of demographics is frequently underestimated [120] and the accuracy of predictions often overestimated; UK demographic models from the 1980s-2000s predicted significantly lower populations in 2020 and beyond, than more recent models [121]. This, in part, is what has led to recent controversial changes to retirement and pensions planning. Several questions emerge around our concept of retirement, our knowledge of the economics and geography of changing demographics, and the role that management design and technology can play in a world where a higher proportion of older people expect to and are expected to make on-going economic contributions, possibly in part time or less heavily loaded positions [122]. There is also a growing demand on the grandparental cohort to support their working age children through babysitting and other family services, which will have important consequences for the labour force and the economy. There are challenges here for policy makers, who will have tough choices to make in the coming years.

**Science and technology challenges:**

- Identifying whether we should change our concepts of career structures and retirement
- Establishing whether we can accurately predict the economics of future demographic structures
- Developing technologies to enable integration of all age groups in society
- Identifying how patterns of work change as age structures change
- Identifying the impact of changing demographics on where people live

### 25. ‘Happy’ Life Expectancy and Government Resource Allocation Decisions

There has been increasing interest in subjective measures of health and wellbeing. Evidence showed that self-rated health predicted the likelihood of dying within a given time period [123], and also measured health in a positive sense [124]. Increased healthy life expectancy, as measured in this way, is one of the overarching outcomes in the Public Health Outcomes Framework, which sets out the government’s vision for the new public health system [125]. A utilitarian would argue that the aim of all government policies, and the organised efforts of society as a whole, should be to maximise positive wellbeing for the maximum number of people for the greatest possible length of time. Such an outcome (which for convenience could be summarised as maximising “happy life expectancy” where “happy” 'is used as a short hand for subjective wellbeing) could be measured in an analogous way to healthy life expectancy by combining population indicator(s) of wellbeing with those of life expectancy. Examples of such indicators have recently been developed by the UK Office for National Statistics (ONS), and the Organisation for Economic Co-operation and Development (OECD) recently produced a guide to measuring subjective wellbeing [126]. A measure of happy life expectancy based on this type of work could be used as a “common currency” across government, and across sectors, to compare the impact of apparently disparate policies and to inform decisions about resource allocation and prioritisation (see also section 14 above on the use wellbeing measures to assess the “State of the Nation”).

**Science and technology challenges:**

- Establishing the best ways for Governments to act, including to allocate public resources and encourage private action, to maximise wellbeing
- Identifying whether such policy tools would be sustainable/ethically acceptable
- Identifying how wellbeing policy objectives could be applied in different sectors of governments
- Determining which measures of wellbeing best represent actual wellbeing reliably
- Determining how to build social and political understanding and consensus around wellbeing metrics

### 26. Decision Making by Autonomous Systems

Technologies that operate with little or distant human control are ever more common [127]. Software that helps machines learn from their environment is rapidly increasing the intelligence of autonomous systems. These technologies promise great benefits, taking on mundane, dangerous and precise tasks. The range of applications is wide, from driverless vehicles, to automated financial trading systems, to autonomous surgical robots. Each of these applications offers diverse benefits and the challenges posed by the technology will vary between sectors [128]. The UK government promised £35 m of extra funding to this area in Autumn 2012, focusing on autonomous robot machines [129]. The UK is a global leader in the development of algorithmic software and has just finished a five-year, £50 m evaluation of opportunities in unmanned airborne vehicles. As a nation at the forefront of the development of these technologies; it is time to lead a much broader public debate about the value of these developments.

**Science and technology challenges:**

- Coordinating and communicating technological assessment of the resilience and safety (human, social, economic) of autonomous systems
- Exploring the social factors that enable trust and or support trustworthiness of autonomous systems, particularly in respect of health, social care and financial systems
- Identifying context-specific factors that affect the reliability of a system
- Identifying the principles and criteria to establish clear instructions for when and how humans should intervene in autonomous systems
- Identify means to enable public discussion of the ethical and technical issues involved in automating decisions

### 27. Managing Demand for Motorised Personal Road Transport

Road transport plays a key role in economic, health and environmental aspects of society. Improving its infrastructure and demand management will result in an increase in road safety and transport efficiency, as well as in a reduction of travel times, congestion, transport costs and carbon emissions [130], [131]. The development of Intelligent Transport Systems (ITS) will provide drivers with real-time information to improve decision-making and facilitate road network management [132], [133]. Examples of such real-time communication could include congestion reports, route guidance, lane departure warnings, blind spot warnings and adaptive cruise control. While most of the technology required for the implementation of traveller information systems already exists, more research is needed on how road users will react to the information and adapt their travel behaviour. Alongside the development and implementation of ITS technology and information delivery systems, economic incentives, such as road pricing schemes, taxes and subsidies could be introduced to improve road network management. However, one of the key implementation challenges remains at the level of public acceptability. In addition, as most of the world’s population now lives in cities, factors affecting urban mobility should also be equally considered: e.g. car sharing schemes and the localised intensification of mass transport.

**Science and technology challenges:**

- Determining how air pollution through road transport can be reduced
- Determining how road-pricing schemes can be implemented efficiently
- Assessing the relative benefits of mass transport vs. individual transport, in terms of accessibility as well as ownership
- Assessing implications of autonomous systems, such as driverless cars
- Encouraging smart network management to reduce emissions and traffic congestion

### 28. Digital Privacy and the Individual

Personal privacy in the digital realm lacks a specific and practical definition, yet it impacts most socio-technical interactions. This gap between knowledge, research and policy needs to be bridged, and consequently a cross-disciplinary research agenda focusing on the balance of technical feasibility and legislative capabilities for digital personal privacy in an international context is required. Important issues include understanding of the impact that growing data repositories (i.e. big data) and potential step changes in computer power (e.g. quantum computing) might have on privacy and both personal and national security [134]; and the impact that human understanding of privacy can have on the behaviour of an individual and mechanisms for communicating privacy [135]. The end goal is establishing and communicating a unified understanding of and potential policy framework for data ownership to augment proposed legislative frameworks [136].

**Science and technology challenges:**

- Identifying mechanisms that enable effective utilisation of available data by private and public entities for commercial and/or national interests
- Understanding the impact growing data repositories and step changes in computer power might have on privacy and on personal and national security
- Assessing the benefits and risks of cloud computing
- Identifying regulatory, industrial and social discrepancies in definitions of digital privacy and their impact on privacy-related threats
- Investigating the potential for mitigating threats through behavioural change and improved public understanding of digital privacy

### 29. Changes in the Technology of Warfare

The means by which war is fought, and the means by which many wider security goals are achieved, are always changing. Throughout history advances in science and technology, or the innovative use of existing technology, have been major factors in those changes. Armed conflict is set within an international legal, moral and ethical framework [137], [138]. These ethical, moral, and legal frameworks also apply to the ways in which war is conducted. Recent technological advances have introduced new paradigms in warfare and national security matters that challenge the existing interpretation of the agreed international legal and ethical norms. Advances in autonomous vehicle use, such as “drone strikes”, have been widely debated with different interpretations [139], [140]. Cyber attacks challenge traditional interpretations of “combatants” and non-combatants” and even what defines war [141]–[143]. There are conflicting views and interpretations across the international community, and a common set of norms needs to be agreed to avoid all actions being judged after the event.

**Science and technology challenges:**

- Developing technical defences to disruption of the interconnectedness of automated and networked modern life
- Identifying and addressing the novel ethical and practical issues that affect the rules of international conflict, particularly in relation to Cyber/AI/Automation/Drones
- Identifying the factors that are changing the nature of deterrence and how this will affect the type and likelihood of attack
- Identifying and developing features that lead to improved resilience
- Determining how technology can contribute towards detection, attribution and response

### 30. Resource Frontiers and Geopolitics

Competition for resources has long been considered as a contributing factor in the cause of war or instability [144]. Access to resources can act as a source of funding and enrichment to the participants, providing personal wealth and thus acting as an incentive to continuing the conflict [145]. New global pressures, most notably climate change, population growth and the need to sustainably manage the world’s rapidly growing demand for energy and water have the potential to create a ‘perfect storm’ of global events [146]. Technology developments over recent decades have re-defined the resources that have strategic importance, and a new era of competition for “economically important raw materials which are subject to a higher risk of supply interruption” (as defined by the EU [147]) is underway. Many nations have set in place, or are developing, resource security strategies, including efficient use, waste minimisation, and resource substitution; the actual approach taken is nation specific. Some States have developed international policy towards ensuring access to strategic resources, such as China’s policy on engagement with Africa [148], [149]; others have focused on harnessing scientific developments to find alternative sources, such as bio-diesel, and fracking [150]. The UK Government has developed an Action Plan on resource security [151].

**Science and technology challenges:**

- Assessing likely future drivers of resource driven interests between countries and regions
- Assessing how Science and Technology could drive alternative technologies that replace current demands
- Determining how extraction technologies might change and the implications for resource availability
- Assess how the capability to exploit new forms of resources or new regional sources might change (e.g. exploitation of the Arctic region)
- Evaluating how can technology and geopolitics interact

## Discussion

We have identified a wide range of issues where public engagement on science and technology might be beneficial or indeed essential. Only a very few of the questions, however, involve ‘technological’ challenges, in the sense of issues requiring a novel technological solution, and corresponding research and development funding. A number of the issues related to aspects of science policy, as opposed to the science itself, such as better coordination, intellectual property, the extent of public understanding and transparency. There were in addition several issues relating to the public “acceptability” of various innovations and, more often than that not, how this was, or was seen to be, a barrier or potential barrier to ‘progress’. This is an area where public engagement has a potentially strong role to play.

Further, a large number of issues related to factors in social change, such as demography or the processes by which local communities respond to global issues [152] and openness. These are not so much science and technology issues in their own right as important emergent social concerns on which science has an important contribution to make to the development of policy responses. A significant subset, for example, relate to incentives and behavioural economics, in contexts such as health, environmental protection and consumption. Similarly, insights from human geography related to phenomena such as ‘glocalisation’ [152] and ‘place-shaping’ [153] would be included here.

In identifying emerging policy issues that require science and technology issues to be addressed rather than research questions in themselves, this exercise has been different in kind to previous exercises using the current methodology [1]. This raises new issues. Any exercise involving experts has limitations [1], [16], [154], the most notable of which is that the output is likely to be heavily determined by the people present at the workshop and that another group might produce a different outcome. This could be due to selection bias in the engagement group, or in response bias to those who were willing to participate. In recognising the need for external validity, the likelihood of bias was minimised in this exercise by drawing on a very large and diverse initial pool of individuals, and by encouraging open discussion and using voting extensively to reduce the impact of dominant individuals. The workshop brought together a broad group from a range of backgrounds, with each individual having an awareness of current and emerging issues in their specialist subject, and many having had previous experience of participating in this type of exercise. Hence, the robustness of the methodology was increased. In total 388 people contributed issues and there were 55 participants in the final workshop, who had a wide range of expertise.

The other important concern is whether the process identifies issues that are genuinely on the horizon (in the sense of emergent, not yet on policy agendas) or whether it picks up more near-field issues that are already gaining policy attention. In that respect, perhaps unsurprisingly, many of the issues have in fact “emerged”, albeit still, in general, being at an early part of the policy cycle. The important factor emerging from this analysis has been the potential public interest in the development of policy on these issues and, linked to it, the research challenges that such potential public interest throws up.

## Conclusion

The intention of this exercise was to identify current and emerging science and technology issues that also have the potential to become issues of public policy or public concern, and thereby might benefit from public engagement and dialogue. This is not to say that these necessarily *will* become issues of policy concern; these are not predictions but observations of emerging issues. Rather, this exercise alerts policy communities to some priorities for policy attention that involve science and technology.

The next steps need to include discussion of these findings with policy communities and analysis of the nature of possible public concerns through public engagement. Many of the issues we have identified will undoubtedly cross departmental and agency boundaries within government, and are notably multidisciplinary. The next stages will, therefore, require the formation of appropriate fora in which policy analysts and specialists from different disciplines can engage, with a view to refining our long list by identifying the specific policy dimensions which would warrant in-depth analysis and dialogue as opposed to those which would simply benefit from the raising of public awareness. This phase would need to address the particular form and timing of engagement, and to identify stakeholder groups whose involvement would be essential to reach outcomes that gain broad assent.

In a UK context, this exercise was also designed with the intention of engaging the UK Parliament and its advisors in horizon scanning activities. Parliament is often overlooked in matters of science and policy [155] and this process was designed both to foster engagement between academics and parliamentarians with an interest in identifying emerging topics of future legislative importance, and to help inform the future work programme of the UK Parliamentary Office of Science and Technology in its role providing parliamentarians with science advice.

## References

[1] Sutherland WJ, Fleishman E, Mascia MB, Pretty P, Rudd MA (2011) Methods for collaboratively identifying research priorities and emerging issues in science and policy. Methods Ecol Evol 2: 238–247.

[2] Jackson R, Barbagallo F, Haste H (2005) Strengths of Public Dialogue on Science-related issues. Critical Review of International Social and Political Philosophy 8: 349–358 10.1080/13698230500187227

[3] Wilsdon J, Willis R (2004) See-through Science: why public engagement needs to move upstream. London: Demos. Available: http://www.greenalliance.org.uk/uploadedFiles/Publications/SeeThroughScienceFinalFullCopy.pdf. Accessed 11 October 2013.

[4] Fiorino DJ (1990) Citizen Participation and Environmental Risk: A Survey of Institutional Mechanisms. Sci Technol Human Values 15: 226–243.

[5] Owens S (2000) Engaging the public: Information and deliberation in environmental policy. Environment and Planning A 32: 1141–1148.

[6] GM Science Review Panel (2003) GM Science Review First Report: An open review of the science relevant to GM crops and food based on interests and concerns of the public. Available: http://www.bis.gov.uk/files/file15655.pdf. Accessed 11 October 2013.

[7] Stirling A (2008) “Opening Up” and “Closing Down”: Power, Participation and Pluralism in the Social Appraisal of Technology. Sci Technol Human Values 33: 262–294.

[8] Fischer F (2009). Democracy and Expertise. Oxford University Press, Oxford. 352 p.

[9] Involve (2007) Democratic technologies? The final report of the Nanotechnology Engagement Group (NEG). Available: http://www.involve.org.uk/wp-content/uploads/2011/03/Democratic-Technologies.pdf. Accessed 11 October 2013.

[10] Shared Practice (2008) Evaluation of Sciencehorizons, Final report. Available: http://www.sciencewise-erc.org.uk/cms/assets/Uploads/Project-files/Sciencehorizons-evaluation-report.pdf. Accessed 11 October 2013.

[11] Linstone HA, Turoff M editors (2002) The Delphi Method: Techniques and Applications, Available: http://is.njit.edu/pubs/delphibook/.Accessed 31 January 2014.

[12] Dalkey N, Helmer O (1963) An Experimental Application of the Delphi Method to the Use of Experts. Management Science, 9, 458–467.

[13] Swor T, Canter L (2011) Promoting environmental sustainability via an expert elicitation process. Environmental Impact Assessment Review, 31, 506–514.

[14] Martin TG, Burgman MA, Fidler F, Kuhnert PM, Low-Choy S, et al (2012) Eliciting Expert Knowledge in Conservation Science. Conservation Biology 26: 29–38 10.1111/j.1523-1739.2011.01806.x 22280323

[15] Rowe G, Wright G (2011) The Delphi technique: past, present and future prospects. Introduction to the special issue. Technological Forecasting and Social Change 78: 1487–1490.

[16] Burgman MA, McBride M, Ashton R, Speirs-Bridge A, Flander L, et al (2011) Expert Status and Performance. PLoS ONE 6(7): e22998 10.1371/journal.pone.0022998 21829574 PMC3146531

[17] Gibbs A (2012) Focus groups and group interviews. Research Methods and Methodologies in Education, 186–192.

[18] Sutherland WJ, Armstrong-Brown S, Armsworth PR, Brereton T, Brickland J, et al (2006) The identification of one hundred ecological questions of high policy relevance in the UK. Journal of Applied Ecology 43: 617–627.

[19] Sutherland WJ, Bailey MJ, Bainbridge IP, Brereton T, Dick JTA, et al (2008) Future novel threats and opportunities facing UK biodiversity identified by horizon scanning. J Appl Ecol 45: 821–833.

[20] Sutherland WJ, Aveling R, Bennun L, Chapman E, Clout M, et al (2012) A Horizon Scan of Global Conservation Issues for 2012. Trends Ecol Evol 27: 12–18. 22133790 10.1016/j.tree.2011.10.011

[21] Pretty J, Sutherland WJ, Ashby J, Auburn J, Baulcombe D, et al (2010) The top 100 questions of importance to the future of global agriculture. International Journal of Agricultural Sustainability 89 (4): 219–236 10.3763/ijas.2010.0534

[22] Sutherland WJ, Bellingan L, Bellingham JR, Blackstock JJ, Bloomfield RM, et al (2011a) A collaboratively-derived science-policy research agenda. PLoS ONE 7(3): e31824 10.1371/journal.pone.0031824 PMC3302883 22427809

[23] Sutherland WL, Goulden C, Bell K, Bennett F, Burrall S, et al. (In Press) 100 Questions: identifying research priorities for poverty prevention and reduction. Journal of Poverty and Social Justice.

[24] Tuck CJ, Hague RMJ, Ruffo M, Ransley M, Adams P (2008) Rapid manufacturing facilitated customization. International Journal of Computer Integrated Manufacturing 21: 245–258 10.1080/09511920701216238

[25] N V (2012) 3D Printing: Difference Engine: The PC all over again? Babbage Science and Technology weblog, 9 September 2012, The Economist. Available: http://www.economist.com/blogs/babbage/2012/09/3d-printing. Accessed 11 October 2013.

[26] Anderson C (2006) The Long Tail: Why the Future of Business Is Selling Less of More. New York: Hyperion. 268 p.

[27] Anderson C (2012) Makers: the new industrial revolution. New York: Crown Business. 272 p.

[28] Sissons A, Thompson S (2012) Three Dimensional Policy, Why Britain needs a policy framework for 3D printing. Big Innovation Centre. Available: http://www.biginnovationcentre.com/Assets/Docs/Reports/3D printing paper_FINAL_15 Oct.pdf. Accessed 11 October 2013.

[29] Rothwell R (1994) Issues in user-producer relations in the innovation process: the role of government. Int J Technol Manag 9: 629–649 10.1504/IJTM.1994.025594

[30] Acemoglu D (2011) Diversity and Technological Progress. National Bureau of Economic Research, Working Papers 16984. Available: http://www.nber.org/papers/w16984.pdf. Accessed 11 October 2013.

[31] Scrase I, Stirling A, Geels FW, Smith A, Van Zwanenberg P (2009) Transformative Innovation: A report to the Department for Environment, Food and Rural Affairs. SPRU - Science and Technology Policy Research, University of Sussex. 67 p.

[32] Department for Business, Innovation & Skills (2011) Innovation and Research Strategy for Growth. The Stationery Office Limited Cm 8239. 104 p. ISBN: 9780101823920. Available: http://www.bis.gov.uk/innovatingforgrowth. Accessed 11 October 2013.

[33] Ofgem (2010) Project Discovery: Options for delivering secure and sustainable energy supplies. Available: http://www.ofgem.gov.uk/Markets/WhlMkts/monitoring-energy-security/Discovery/Documents1/Project_Discovery_FebConDoc_FINAL.pdf. Accessed 11 October 2013.

[34] Engineering and Physical Science Research Council (2011) Details of Grant: The Autonomic Power System. Available: http://gow.epsrc.ac.uk/NGBOViewGrant.aspx?GrantRef=EP/I031650/1. Accessed 11 October 2013.

[35] Willis R, Eyre N (2011) Demanding Less: Why we need a new politics of energy. Green Alliance. Available: http://www.green-alliance.org.uk/grea_p.aspx?id=6177. Accessed 11 October 2013.

[36] HM Government (2009) The UK Low Carbon Transition Plan: National Strategy for Climate and Energy. Norwich: The Stationary Office. Available: http://www.official-documents.gov.uk/document/other/9780108508394/9780108508394.pdf. Accessed 11 October 2013.

[37] Pudjianto D, Ramsay C, Strbac G (2007) Virtual power plant and system integration of distributed energy resources. Renewable Power Generation, IET 1: 10–16.

[38] Hargreaves T, Nye M, Burgess J (2013) Keeping energy visible? Exploring how householders interact with feedback from smart energy monitors in the longer term. Energy Policy 52: 126–134.

[39] National Endowment for Science and the Arts (Nesta) (2013) Dynamic Demand: The Challenge of shifting peak electricity demand. Available: http://www.nesta.org.uk/library/documents/Challenge_shifting_electricity.pdf. Accessed 11 October 2013.

[40] Hammond G, Pearson P (2013) Challenges of the transition to a low carbon, more electric future: From here to 2050. Energy Policy 52: 1–9.

[41] Wilks L, Billsberry J (2007) Should we do away with teleworking? An examination of whether teleworking can be defined in the new world of work. New Technology, Work and Employment 22: 168–177.

[42] White P, Christodoulou G, Mackett R, Titheridge H, Thoreau R, et al.. (2010) Impacts of teleworking on sustainability and travel. In: Manzi T, Lucas K, Lloyd Jones T, Allen J, editors. (2010) Social Sustainability in Urban Areas: Communities, Connectivity and the Urban Fabric. London: Earthscan, 141–154.

[43] Infrastructure UK (2011) National Infrastructure Plan 2011. HM Treasury. Available: http://cdn.hm-treasury.gov.uk/national_infrastructure_plan291111.pdf. Accessed 11 October 2013.

[44] House of Lords Science and Technology Committee (2011) 2nd Report of Session 2010–12: Behaviour Change. HL Paper 179, London: The Stationery Office. http://www.publications.parliament.uk/pa/ld201012/ldselect/ldsctech/179/179.pdf. Accessed 11 October 2013.

[45] Parliamentary Office of Science and Technology (2012) Energy Use Behaviour Change. POSTnote 417. Available: http://www.parliament.uk/business/publications/research/briefing-papers/POST-PN-417.

[46] FAO WFP, IFAD (2012) The State of Food Insecurity in the World 2012. Economic growth is necessary but not sufficient to accelerate reduction of hunger and malnutrition. Rome, FAO. Available: http://www.fao.org/docrep/016/i3027e/i3027e.pdf. Accessed 11 October 2013. 10.3945/an.112.003343 PMC3648734 23319131

[47] Godfray HCJ, Beddington JR, Crute IR, Haddad L, Lawrence D, et al (2010) Food security: the challenge of feeding 9 billion people. Science 327 (5967): 812–818 10.1126/science.1185383 20110467

[48] Foresight (2011) The Future of Food and Farming. London, Government Office of Science. Available: http://www.bis.gov.uk/assets/foresight/docs/food-and-farming/11-546-future-of-food-and-farming-report.pdf. Accessed 11 October 2013.

[49] Garnett T, Appleby MC, Balmford A, Bateman IJ, Benton TG, et al (2013) Sustainable Intensification in Agriculture: Premises and Policies. Science 341: 33–34. 23828927 10.1126/science.1234485

[50] Steffen W, Grinevald J, Crutzen P, McNeill J (2012) The Anthropocene: conceptual and historical perspectives. Philos Trans R Soc Lond A 369 (198): 842–867 10.1098/rsta.2010.0327 21282150

[51] Stafford Smith M, Horrocks L, Harvey A, Hamilton C (2011) Rethinking adaptation for a 4°C world. Philos Trans R Soc Lond A 369: 196–216. 10.1098/rsta.2010.0277 21115520

[52] O’Brien K (2012) Global environmental change II: From adaptation to deliberate transformation. Progr Hum Geog 36: 667–676 10.1177/0309132511425767

[53] Rockström J, Steffen W, Noone K, Persson A, Chapin FS, et al (2009) Planetary boundaries: exploring the safe operating space for humanity. Ecology and Society 14: 32.

[54] Scheffer M, Carpenter SR, Lenton TM, Bascompte J, Brock W, et al (2012) Anticipating Critical Transitions. Science 338: 344–348 10.1126/science.1225244 23087241

[55] Lenton TM, Ciscar JC (2013) Integrating tipping points into climate impact assessments. Clim Change 117: 585–597 10.1007/s10584-012-0572-8

[56] Royal Commission on Environmental Pollution (2010) Adapting Institutions to Climate Change. Twenty-eighth Report. London: The Stationery Office.

[57] Adger WN, Arnell NW, Tompkins EL (2005) Successful adaptation to climate change across scales. Glob Environ Change 15: 77–86.

[58] Department for Environment, Food and Rural Affairs (2009) UK Climate Projections. Available: http://ukclimateprojections.defra.gov.uk. Accessed 11 October 2013.

[59] Department for Environment, Food and Rural Affairs (2012) Summary of the Key Findings from the UK Climate Change Risk Assessment 2012. Available: http://www.defra.gov.uk/sac/files/SAC1215-CCRA-Paper-Annex-1-Key-Findings.pdf. Accessed 11 October 2013.

[60] HM Government (2013) The National Adaptation Programme, HMSO London. Available: https://www.gov.uk/government/publications/adapting-to-climate-change-national-adaptation-programme. Accessed 11 October 2013.

[61] Royal Society (2009) Geoengineering the climate: science, governance and uncertainty. Policy document 10/09. London: The Royal Society. Available: http://royalsociety.org/uploadedFiles/Royal_Society_Content/policy/publications/2009/8693.pdf. Accessed 11 October 2013.

[62] Government Accountability Office (2011) Climate Engineering: Technical Status, Future Directions, and Potential Responses. Centre for Science, Technology, and Engineering, GAO-11-71. Available: http://www.gao.gov/assets/330/322208.pdf. Accessed 11 October 2013.

[63] Jones C, Williamson P, Haywood J, Lowe J, Wiltshire A, et al. (2013) LWEC Geoengineering Report: A forward look for UK research on climate impacts of geoengineering. Available: http://www.lwec.org.uk/sites/default/files/attachments_page/GE_Forward_Look_FINAL_02 Sept 2013.pdf. Accessed 11 October 2013.

[64] Rayner S, Redgewell C, Savulescu J, Pidgeon N, Kruger T (2010) Draft principles for the conduct of geoengineering research. Written Evidence Memoranda 42 and 44 in [59].

[65] House of Commons Science and Technology Committee (2010) The Regulation of Geoengineering. Fifth Report of Session 2009–10; HC 221. London: The Stationery Office Limited. Available: http://www.publications.parliament.uk/pa/cm200910/cmselect/cmsctech/221/221.pdf. Accessed 11 October 2013.

[66] Parliamentary Office of Science and Technology (2011) Landscapes of the Future. POSTnote 380 Available: http://www.parliament.uk/business/publications/research/briefing-papers/POST-PN-380. Accessed 11 October 2013.

[67] UK National Ecosystem Assessment (2011) The UK National Ecosystem Assessment: Synthesis of the Key Findings. Cambridge: UNEP-WCMC. Available: http://uknea.unep-wcmc.org/LinkClick.aspx?fileticket=ryEodO1KG3k=&tabid=82. Accessed 11 October 2013.

[68] Government Office for Science (2010) Foresight Land Use Project: making the most of land in the 21st century. Available: http://www.bis.gov.uk/assets/foresight/docs/land-use/luf_report/8614-bis-land_use_futures_exec_summ-web.pdf. Accessed 11 October 2013.

[69] Tilman D, Socolow R, Foley JA, Hill J, Larson E, et al (2009) Beneficial Biofuels: The Food, Energy, and Environment Trilemma. Science 325: 270–271 10.1126/science.1177970 19608900

[70] Natural Capital Committee (2013) The State of Natural Capital: Towards a framework for measurement and valuation. Available: http://www.defra.gov.uk/naturalcapitalcommittee/files/State-of-Natural-Capital-Report-2013.pdf. Accessed 11 October 2013.

[71] Risk and Regulation Advisory Council (2009) The risk landscape, interactions that shape response to public risk. Available: http://webarchive.nationalarchives.gov.uk/20100104183913/http://www.berr.gov.uk/files/file51457.pdf. Accessed 11 October 2013.

[72] Rocks SA, Owen R, Pollard SJ, Dorey RA, Harrison PTC, et al.. (2009) Risk assessment of manufactured nanomaterials. In: Lead J, Smith E, editors. Environmental and Human Health Effects of Nanoparticles. 389–421. Wiley-Blackwell.

[73] Department for Environment, Food and Rural Affairs (2011) Greenleaves III – Guidelines for Environmental Risk Assessment and Managemen*t* Available: https://www.gov.uk/government/uploads/system/uploads/attachment_data/file/69450/pb13670-green-leaves-iii-1111071.pdf. Accessed 11 October 2013.

[74] Taylor CM, Pollard SJT, Angus AJ, Rocks SA (2013) Better by design: rethinking interventions for better environmental regulation. Sci Total Environ 447: 488–499. 23410870 10.1016/j.scitotenv.2012.12.073

[75] HM Treasury (2004) The Orange Book: Management of Risk - Principles and Concepts. Available: https://www.gov.uk/government/uploads/system/uploads/attachment_data/file/220647/orange_book.pdf. Accessed 11 October 2013.

[76] Collingridge D (1980) The Social Control of Technology. London, Frances Pinter. 200 p.

[77] Douron M (2010) U-Shaped Dose-Response Curves: Implications for Risk Characterization of Essential Elements and Other Chemicals. Journal of Toxicology and Environmental Health A 73 2–3: 181–186. 10.1080/15287390903340450 20077289

[78] Kortenkamp A, Martin O, Faust M, Evans R, McKinlay R, et al. (2012) State of the art assessment of endocrine disrupters, Project contract number 070307/2009/550687/SER/D3 Directorate-General for the Environment, European Commission. Available: http://ec.europa.eu/environment/chemicals/endocrine/pdf/sota_edc_final_report.pdf. Accessed 12 April 2014.

[79] Holt JC (1964) How Children Fail. New York: Pitman. 181p.

[80] Matterson C, Holman J (2012) Informal Science Learning Review: Reflections from the Wellcome Trust. London: Wellcome Trust. Available: http://www.wellcome.ac.uk/stellent/groups/corporatesite/@msh_peda/documents/web_document/wtp040859.pdf. Accessed 11 October 2013.

[81] Cusumano MA (2013) Are the costs of ‘free’ too high in online education? Communications of the ACM 56: 26–28.

[82] Swan K (2002) Building learning communities in online courses: the importance of interaction. Education, Communication & Information 2: 23–49.

[83] Woo Y, Reeves TC (2007) Meaningful interaction in web-based learning: A social constructivist interpretation. The Internet and Higher Education 10: 15–25.

[84] House of Commons Public Administration Select Committee (2012) Strategic Thinking in Government: Without National Strategy, can viable Government strategy emerge; Twenty-Fourth Report of Session 2010–12, HC 1625, London: The Stationery Office.

[85] Kahneman D, Diener E, Schwarz N, editors (2003) Well-being: The foundations of hedonic psychology. New York, NY: Russel Sage Foundation. 597p.

[86] Parliamentary Office of Science and Technology (2012) Measuring National Wellbeing. POSTnote 421. Available: http://www.parliament.uk/business/publications/research/briefing-papers/POST-PN-421.

[87] Ho P (2012) Coping with complexity. In: McKinsey and Company. Government designed for new times. 82–84. Available: http://www.google.co.uk/url?sa=t&rct=j&q=&esrc=s&source=web&cd=1&ved=0CDMQFjAA&url=http%3A%2F%2Fwww.mckinsey.com%2Ffeatures%2Fgovernment_designed_for_new_times%2F~%2Fmedia%2F1F14299ED7E141C3831127523960DA69.ash&ei=71pEUsXABumo0QXrmIHgCQ&usg=AFQjCNH2rb3ojwdmDZ5MZSAowcD3uMZttA&bvm=bv.53217764,d.d2k. Accessed 11 October 2013.

[88] Rosenhead J (2001) Complexity theory and management practice. Working paper series LSEOR 98.25, London School of Economics.

[89] Munda (2000) Conceptualising and Responding to Complexity. Environmental valuation in Europe: Policy Research Brief Number 2. ISBN 186190 0821. Available: http://www.macaulay.ac.uk/serp/research/eve/publ.htm. Accessed 11 October 2013.

[90] Ramalingam B, Jones H, Reba T, Young J (2008) Exploring the science of complexity: Ideas and implications for development and humanitarian efforts. ODI working paper 285. Overseas Development Institute. Available: http://www.odi.org.uk/sites/odi.org.uk/files/odi-assets/publications-opinion-files/833.pdf. Accessed 11 October 2013.

[91] Snowden D, Boone M (2007) A Leader’s Framework for Decision Making. Harvard Buisness Review 2007: 69–76. 18159787

[92] Wright C, Kiparoglou V, Williams M, Hilton J (2012) A Framework for Resilience Thinking. New Challenges in Systems Engineering and Architecting. Conference on Systems Engineering Research (CSER 2012: 45–52) St. Louis, MO. Cihan H. Dagli, Editor in Chief. Missouri University of Science and Technology.

[93] Jones H (2011) Taking responsibility for complexity: How implementation can achieve results in the face of complex problems. ODI Working Paper 330, June. London: Overseas Development Institute. Available: http://www.odi.org.uk/sites/odi.org.uk/files/odi-assets/publications-opinion-files/6485.pdf. Accessed 11 October 2013.

[94] Ostrom E, Hess C, editors (2006) Understanding Knowledge as a Commons: From Theory to Practice. The MIT Press, Cambridge, Massachusetts. 384 p.

[95] Council for Science and Technology (2009) A National Infrastructure for the 21^st^ Century. Available: http://www.bis.gov.uk/assets/cst/docs/files/whats-new/09-1631-national-infrastructure. Accessed 11 October 2013.

[96] Infrastructure UK (2012) National Infrastructure Plan: update 2012. HM Treasury. Available: http://www.hm-treasury.gov.uk/d/national_infrastructure_plan_051212.pdf. Accessed 11 October 2013.

[97] Nutt D (2012) Drugs without the hot air. Cambridge: UIT Cambridge. 352 p.

[98] HM Government (2012) Civil Service Reform. Available: http://www.civilservice.gov.uk/wp-content/uploads/2012/06/Civil-Service-Reform-Plan-acc-final.pdf. Accessed 11 October 2013.

[99] Government Science and Engineering (2013) The Future of the Civil Service: Making the most of Scientists and Engineers in Government, a review of the science and engineering profession in the Civil Service. Available: http://www.bis.gov.uk/assets/goscience/docs/r/bis-13-594-review-science-engineering-in-civil-service.pdf. Accessed 11 October 2013.

[100] UK Parliament (2013) Public Engagement in Policymaking. Available: http://www.publications.parliament.uk/pa/cm201213/cmselect/cmpubadm/writev/publicpolicy/m13.htm. Accessed 11 October 2013.

[101] HM Government (2012) Civil Service Reform – Part 2– Improving policy making capability. Civil Service. Available: http://www.civilservice.gov.uk/reform/part-2-improving-policy-making-capability. Accessed 11 October 2013.

[102] Parliamentary Office of Science and Technology (2013) Managing Online Identity. POSTnote 434. Available: http://www.parliament.uk/business/publications/research/briefing-papers/POST-PN-434. Accessed 11 October 2013.

[103] Zacharzewsk A, Mulcare C, Latta S (2013) The benefits and risks of digital engagement. Sciencewise-ERC. Available: http://www.sciencewise-erc.org.uk/blog/?p=879. Accessed 11 October 2013.

[104] HM Government (No Date) UK Resilience and communicating risk (Guidance). Available: https://www.gov.uk/government/uploads/system/uploads/attachment_data/file/60907/communicating-risk-guidance.pdf. Accessed 11 October 2013.

[105] Meissner A, Luckenbach T, Risse T, Kirste T, Kirchner H (2002) Design Challenges for an Integrated Disaster Management Communication and Information System. Available: http://www.l3s.de/~risse/pub/P2002-01.pdf. Accessed 11 October 2013.

[106] Baylis M, Githeko AK (2006) Infectious Diseases: preparing for the future. The Effects of Climate Change on Infectious Diseases of Animals. Foresight Office of Science and Innovation, report T7.3. Available: http://www.bis.gov.uk/assets/foresight/docs/infectious-diseases/t7_3.pdf. Accessed 11 October 2013.

[107] Fisher MC, Henk DA, Briggs CJ, Brownstein JS, Madoff LC, et al (2012) Emerging fungal threats to animal, plant and ecosystem health. Nature 484: 186–194 10.1038/nature10947 22498624 PMC3821985

[108] MacLean D, Yoshida K, Edwards A, Crossman L, Clavijo B, et al.. (2013) Crowdsourcing genomic analyses of ash and ash dieback – power to the people, Gigascience, 2(2), doi:10.1186/2047-217X-2-2. 10.1186/2047-217X-2-2 PMC3626535 23587306

[109] Ginsberg J, Mohebbi MH, Patel RS, Brammer L, Smolinski MS, et al (2009) Detecting influenza epidemics using search engine query data. Nature 457: 1012–1014 10.1038/nature07634 19020500

[110] Department of Health (2012) Antibiotic resistance poses alarming threat. Available: https://www.gov.uk/government/news/antibiotic-resistance-poses-alarming-threat. Accessed 11 October 2013.

[111] World Health Organisation (2013) Drug Resistance. Available: http://www.who.int/topics/drug_resistance/en/. Accessed 11 October 2013.

[112] DoH 2013 UK Five Year Antimicrobial Resistance Strategy 2013 to 2018. Available: https://www.gov.uk/government/uploads/system/uploads/attachment_data/file/244058/20130902_UK_5_year_AMR_strategy.pdf. Accessed 11 October 2013.

[113] Carlet J, Pittet D (2013) Access to antibiotics: a safety and equity challenge for the next decade. Antimicrob Resist Infect Control, 2 (1), doi:10.1186/2047-2994-2-1. 10.1186/2047-2994-2-1 PMC3599140 23305311

[114] Bech M, Christiansen T, Khoman E, Lauridsen J, Weale M (2011) Ageing and health care expenditure in EU-15. European Journal of Health Economics 12: 469–478. 20574765 10.1007/s10198-010-0260-4

[115] Hex N, Bartlett C, Wright D, Taylor M, Varley D (2012) Estimating the current and future costs of Type 1 and Type 2 diabetes in the UK, including direct health costs and indirect societal and productivity costs. Diabet Med. 29: 855–62. 10.1111/j.1464-5491.2012.03698.x 22537247

[116] Nolte E, Knai C, McKee M (2008) Managing chronic conditions: Experience in eight countries. WHO European Observatory on Health Systems and Policies Studies Series No 15. Available: http://www.euro.who.int/__data/assets/pdf_file/0008/98414/E92058.pdf. Accessed 11 October 2013.

[117] Department of Business Innovation and Skills (2011) Strategy for UK Life Sciences. Available: https://www.gov.uk/government/uploads/system/uploads/attachment_data/file/32457/11-1429-strategy-for-uk-life-sciences.pdf. Accessed 11 October 2013.

[118] de la Maisonneuve C, Oliveira Martins J (2013) Public Spending on health and long-term care: a new set of projections. OECD Economic Policy Papers No 6 2013 ISSN 2226583X.

[119] Ham C, Dixon A, Brooke B (2012) Transforming the Delivery of Health and Social Care: the case for fundamental change. The Kings Fund. 10.3399/bjgp13X668104 PMC3662430 23735385

[120] Willetts D (2011) The Pinch: How the Baby Boomers Took Their Children's Future - And Why They Should Give it Back. London: Atlantic Books.

[121] Parliamentary Office of Science and Technology (2013) Uncertainty in Population Projections. POSTnote 438. Available: http://www.parliament.uk/business/publications/research/briefing-papers/POST-PN-438. Accessed 11 October 2013.

[122] Parliamentary Office of Science and Technology (2011) An Aging Workforce. POSTnote 391 Available: http://www.parliament.uk/business/publications/research/briefing-papers/POST-PN-391. Accessed 11 October 2013.

[123] Idler E, Benyamini Y (1997) Self-rated health and mortality: A review of twenty-seven community studies. Journal of Health and Social Behaviour 38: 21–37. 9097506

[124] Kelly S, Baker A, Gupta S (2000) Healthy life expectancy in Great Britain, 1980–96, and its use as an indicator in UK Government strategies. Health Stat Q 7: 32–37.

[125] Department of Health (2012) Public Health Outcomes Framework update: Improving outcomes and supporting transparency, 2013–2016. Available: https://www.gov.uk/government/publications/public-health-outcomes-framework-update. Accessed 11 October 2013.

[126] Organisation for Economic Co-operation & Development (2013) OECD Guidelines on Measuring Subjective Well-being. OECD Publishing, Paris. Available: 10.1787/9789264191655-en Accessed 11 October 2013.

[127] Aerospace, Aviation & Defence Knowledge Transfer Network (2012) Autonomous Systems: Opportunities and Challenges for the UK. Available: https://connect.innovateuk.org/web/autonomous-systems-ntc. Accessed 11 October 2013.

[128] Royal Academy of Engineering (2009) Autonomous Systems: Social, Legal and Ethical Issues. London: The Royal Academy of Engineering. Available: http://www.raeng.org.uk/societygov/engineeringethics/pdf/Autonomous_Systems_Report_09.pdf. Accessed 11 October 2013.

[129] Willetts D (2013) Eight Great Technologies. The Policy Exchange. Available: http://www.policyexchange.org.uk/publications/category/item/eight-great-technologies. Accessed 11 October 2013.

[130] Department for Transport (2008) Delivering a sustainable transport system: Main report. London: Department for Transport. Available: http://webarchive.nationalarchives.gov.uk/20081230052656/http://www.dft.gov.uk/about/strategy/transportstrategy/dasts/dastsreport.pdf. Accessed 11 October 2013.

[131] Hickman R, Ashiru O, Banister D (2010) Transport and climate change: simulating the options for carbon reduction in London. Transport Policy 17: 110–125 10.1016/j.tranpol.2009.12.002

[132] Parliamentary Office of Science and Technology (2009) Intelligent Transport Systems. POSTnote 322. Available at: http://www.parliament.uk/briefing-papers/POST-PN-322.pdf. Accessed 11 October 2013.

[133] Institution of Engineering and Technology and Intelligent Transport Systems (UK) (2011) Can we really do more at less cost with the UK road network? Available: http://www.its-uk.org.uk/filelibrary/file/more-for-less.pdf. Accessed 11 October 2013.

[134] Kshetri N, Murugesan S (2013) Cloud Computing and EU Data Privacy Regulations. Computer 46: 86–89 10.1109/MC.2013.86

[135] Cadogan RA (2004) An imbalance of power: the readability of internet privacy policies. Journal of Business & Economics Research 2: 49–62.

[136] European Commission (2012) Proposal for a Regulation of the European Parliament and of the Council on the protection of individuals with regard to the processing of personal data and on the free movement of such data (General Data Protection Regulation), SEC(2012) 72 (final). Available: http://ec.europa.eu/justice/data-protection/document/review2012/com_2012_11_en.pdf. Accessed 11 October 2013.

[137] Cook M (2001) Ethical issues in War, An overview. In: Cerami JR, Holcomb JF, editors. U.S. Army War College guide to strategy. Strategic Studies. 19–30.

[138] United Nations (2013) Charter of the United Nations. Available: http://Www.Un.Org/En/Documents/Charter/Chapter7.Shtml. Accessed 11 October 2013.

[139] Brooke-Holland L (2013) Unmanned Aerial Vehicles (drones): an introduction – UK House of Commons Library Standard Note SN06493. Available: http://www.parliament.uk/briefing-papers/SN06493/unmanned-aerial-vehicles-(drones)-an-introduction. Accessed 11 October 2013.

[140] Thorp A (2011) Drone attacks and the killing of Anwar al-Awlaqi: legal issues – UK House of Commons Library Standard Note SN06165. Available: http://www.parliament.uk/briefing-papers/SN06165/drone-attacks-and-the-killing-of-anwar-al-awlaqi-legal-issues. Accessed 11 October 2013.

[141] Hathaway OA, Crootof R, Levitz P, Nix H, Nowlan A, et al.. (2012) The Law of Cyber-Attack. California Law Review 100: 817–885. Accessed 11 October 2013.

[142] Schmitt MN (2012) “Attack” as a Term of Art in International Law: The Cyber Operations Context. 4th International Conference on Cyber Conflict. Czosseck C, Ottis R, Ziolkowski K, editors. NATO CCD COE Publications, Tallinn. http://www.ccdcoe.org/publications/2012proceedings/5_2_Schmitt_AttackAsATermOfArt.pdf. Accessed 11 October 2013.

[143] Lewis JA (2010) A Note on the Laws of War in Cyberspace. Center for Strategic and International Studies. Available: http://csis.org/files/publication/100425_Laws%20of%20War%20Applicable%20to%20Cyber%20Conflict.pdf. Accessed 11 October 2013.

[144] Berdal M, Keen D (1997) Violence and Economic Agendas in Civil Wars: Some Policy Implications. Millennium: Journal of International Studies 26: 795–818.

[145] Haida H (2012) GSDRC Topic Guide on Conflict. Available: http://www.gsdrc.org/index.cfm?objectid=4A0C23DB-14C2-620A-27D1F2B5EF89AA1A. Accessed 11 October 2013.

[146] Beddington J (2009) Food, energy, water and the climate: a perfect storm of global events? Available: http://www.bis.gov.uk/assets/goscience/docs/p/perfect-storm-paper.pdf. Accessed 11 October 2013.

[147] European Commission (No date) defining “critical” raw materials. Available: http://ec.europa.eu/enterprise/policies/raw-materials/critical/index_en.htm. Accessed 11 October 2013.

[148] Basu N (2013) China and Africa: Is the Honeymoon Over? Foreign Policy Journal. Available: http://www.foreignpolicyjournal.com/2013/04/03/china-and-africa-is-the-honeymoon-over/. Accessed 11 October 2013.

[149] Iyasu AA (2013) China’s Non-Interference Policy and Growing African Concerns http://africanarguments.org/2013/07/18/china%E2%80%99s-non-interference-policy-and-growing-african-concerns/.Accessed 11 October 2013.

[150] Koranyi D, editor. (2011) Transatlantic Energy Futures: Strategic Perspectives on Energy Security, Climate Change, and New Technologies in Europe and the United States. Washington, DC: Center for Transatlantic Relations. Available: http://transatlantic.sais-jhu.edu/publications/books/Transatlantic_Energy_Futures/Transatlantic_Energy_Futures.pdf. Accessed 11 October 2013.

[151] Department for Environment, Food and Rural Affairs (2012) A Review of National Resource Strategies and Research. Available: https://www.gov.uk/government/uploads/system/uploads/attachment_data/file/69526/pb13722-national-resource-strategies-review.pdf. Accessed 11 October 2013.

[152] Roudometof V (2005) Translationalism, Cosmopolitanism, and Glocalization. Current Sociology 53: 113–135.

[153] Lyons M (2007) Place-shaping: a shared ambition for the future of local government. The Lyons Inquiry into Local Government (Executive Summary). London, The Stationery Office. Available: http://www.webarchive.org.uk/wayback/archive/20070329120000/http://www.lyonsinquiry.org.uk/docs/final-exec.pdf. Accessed 11 October 2013.

[154] Sutherland WJ (2013) Review by quality not quantity for better policy. Nature 503, 167. 10.1038/503167a 24226852

[155] Tyler C (2013) Scientific Advice in Parliament. In: Doubleday R, Wilsdon J, editors. Future directions for Scientific Advice in Whitehall. London: Institute for Government/CSaP/SPRU/Alliance for Useful Evidence 115–120. Available: http://www.csap.cam.ac.uk/media/uploads/files/1/fdsaw.pdf. Accessed 11 October 2013.

